# The prognostic value of hemoglobin-to-albumin ratio in critically ill patients with atrial fibrillation: A retrospective cohort study

**DOI:** 10.1097/MD.0000000000048211

**Published:** 2026-04-24

**Authors:** Yinghan Sun, Yuxiao Zhai, Hongyi Cai, Chunyang Lv

**Affiliations:** a Department of Cardiology, The Fourth Affiliated Hospital of Harbin Medical University, Harbin, China; b Department of Critical Care Medicine, Bao’an District People’s Hospital, Shenzhen, China.

**Keywords:** atrial fibrillation, biomarker, hemoglobin-to-albumin ratio, intensive care unit, mortality, prognosis

## Abstract

Atrial fibrillation (AF) is common in critically ill patients and contributes to poor outcomes. Existing risk scores provide limited prognostic value for overall mortality. The hemoglobin-to-albumin ratio (HAR), reflecting hematologic and nutritional-inflammatory status, may serve as a novel prognostic biomarker in this setting. We performed a retrospective cohort study using the Medical Information Mart for Intensive Care IV database (version 3.1). Adult intensive care unit patients with AF were included, excluding those with multiple admissions, hospital stay <24 hours, or missing laboratory data. The primary outcome was 28-day all-cause mortality, and the secondary outcome was 360-day mortality. Patients were stratified by HAR quartiles. Kaplan–Meier analysis, Cox proportional hazards models, and restricted cubic splines were applied. Subgroup analyses assessed consistency across clinical strata. A total of 7801 patients were included (median age 75 years; 40.9% male). The 28- and 360-day mortality rates were 20.1% and 25.9%, respectively. Higher HAR was associated with increased mortality (log-rank *P* < .001). In fully adjusted Cox models, HAR independently predicted 28-day mortality (hazard ratio: 1.07, 95% confidence interval: 1.03–1.11) and 360-day mortality (hazard ratio: 1.07, 95% confidence interval: 1.04–1.11). Restricted cubic splines showed an approximately linear relationship. Subgroup analyses indicated stronger associations in older patients, those not receiving beta-blockers, and in amiodarone users at 360 days. HAR is an independent predictor of short- and long-term mortality in critically ill AF patients. As a simple and routinely available biomarker, HAR may enhance risk stratification beyond conventional clinical tools. Further prospective and multicenter studies are warranted to validate these findings.

## 1. Introduction

Atrial fibrillation (AF) is the most common sustained arrhythmia encountered in clinical practice and is strongly associated with increased morbidity and mortality,^[1,2]^ particularly in critically ill populations.^[3–6]^ Accurate risk stratification remains a cornerstone for improving prognosis and tailoring therapeutic strategies in AF patients admitted to intensive care units (ICUs). Traditional risk scores, such as the CHA_2_DS_2_-VASc and HAS-BLED scores, mainly address thromboembolic and bleeding risk but provide limited predictive value for short- and long-term all-cause mortality.^[7–9]^ Hence, there is an urgent need to identify novel, easily obtainable biomarkers that can refine prognostic assessment in this high-risk group.

Hemoglobin (Hgb) and albumin are routinely measured laboratory parameters that reflect systemic oxygen-carrying capacity, nutritional status, and systemic inflammation. Previous studies have shown that both anemia and hypoalbuminemia independently predict adverse outcomes in cardiovascular disease, including heart failure (HF), acute myocardial infarction, and AF-related complications.^[10–12]^ Recently, the hemoglobin-to-albumin ratio (HAR) has emerged as a novel composite index integrating hematologic and nutritional-inflammatory status. Although HAR has been investigated primarily in oncologic and surgical settings as a predictor of survival,^[13,14]^ its relevance in cardiovascular and critical care medicine has received limited attention. Given that systemic inflammation and nutritional status are closely linked to outcomes in patients with AF, we sought to evaluate the prognostic value of HAR in this population.^[15]^

Leveraging the large-scale Medical Information Mart for Intensive Care IV (MIMIC-IV) database, we conducted an exploratory analysis to examine the association between HAR and both short- and long-term mortality in critically ill patients with AF. Specifically, we aimed to assess the predictive value of HAR for 28-day all-cause mortality as the primary outcome and 360-day all-cause mortality as the secondary outcome. By systematically examining this novel biomarker in a real-world ICU population, our study aimed to provide new insights into risk stratification and long-term prognosis of AF patients. We hypothesized that a higher HAR would be independently associated with increased short- and long-term all-cause mortality in critically ill patients with AF.

## 2. Materials and methods

### 2.1. Study population

**Figure 1.**
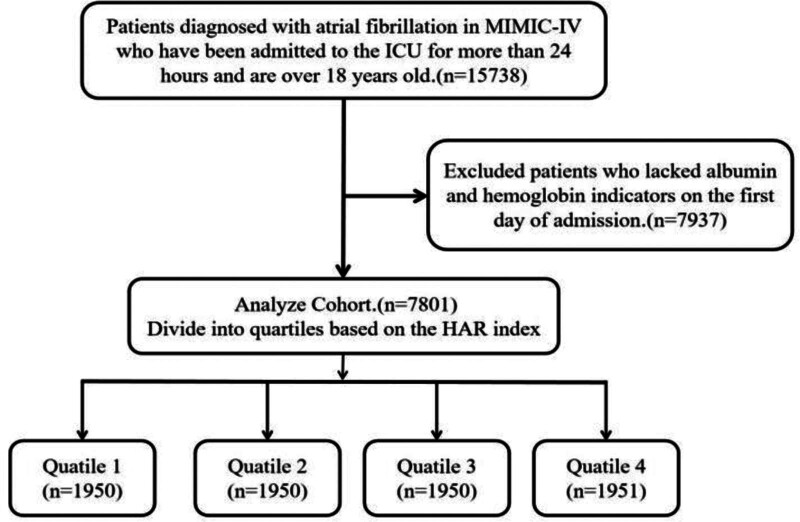
Flowchart of the study population selection process. This figure illustrates the step-by-step exclusion criteria and the final enrollment of 7801 critically ill patients with atrial fibrillation from the MIMIC-IV database. HAR = hemoglobin-to-albumin ratio, ICU = intensive care unit, MIMIC-IV = Medical Information Mart for Intensive Care IV.

This retrospective study investigated health-related data obtained from the MIMIC-IV (version 3.1) database, which is a common and large database that was developed and managed by the MIT Computational Physiology Laboratory.^[16,17]^ This database comprises extensive and high-quality medical records of patients who were admitted to the intensive critical care units of the Beth Israel Deaconess Medical Center. One author (Yinghan Sun) complied with the requirements for access to the database and was responsible for the data extraction. Patients who were diagnosed with AF were enrolled in this study according to the International Classification of Diseases, 9th and 10th Revision. The exclusion criteria were as follows: patients aged <18 years or not at their first admission; patients with multiple admissions for AF, for whom only the first admission data were extracted; patients with a hospital length of stay of <24 hours; and patients lacking Hgb and albumin laboratory data within 24 hours after admission. Finally, a total of 7801 patients were enrolled in this study and grouped into 4 groups based on the quartiles of the HAR index (Fig. 1).

### 2.2. Data collection

The software PostgreSQL (version 13.7.2; PostgreSQL Global Development Group) and Navicat Premium (version 16; PremiumSoft CyberTech Ltd, Hong Kong, China) were used to extract information using a running Structured Query Language. The extraction of potential variables was divided into 6 groups: demographics, including age, sex, race, and weight; comorbidities, including cardiac valvulopathy, diabetes, hypertension, HF, myocardial infarction, and chronic obstructive pulmonary disease (COPD); laboratory indicators, including albumin, Hgb, white blood cell count (WBC), red blood cell distribution width, sodium, potassium, calcium, magnesium, lactate dehydrogenase, glucose, international normalized ratio (INR), and creatinine; severity-of-illness scores at admission, including the Charlson Comorbidity Index, the Simplified Acute Physiology Score II (SAPS II), and the Sequential Organ Failure Assessment (SOFA); vital signs, including heart rate, respiratory rate (RR), peripheral oxygen saturation, and mean noninvasive blood pressure; and medication use, including insulin, heparin, warfarin, amiodarone, beta-blockers, and new oral anticoagulants/direct oral anticoagulants (NOACs/DOACs). Follow-up began on the date of admission and ended on the date of death. The HAR index is calculated by dividing Hgb (g/dL) by albumin (g/dL). All laboratory variables and disease severity scores were extracted from the data generated within the first 24 hours after the patient entered the ICU. To avoid possible bias, variables were excluded if they had more than 20% missing values. Variables with <20% missing data were reported for missing items and proportions (Table S2, Supplemental Digital Content, https://links.lww.com/MD/R653), and multiple imputation was performed using the “mice” package in R software (version 4.0.2; R Foundation for Statistical Computing, Vienna, Austria) with a random forest algorithm (trained by other non-missing variables).

### 2.3. Clinical outcomes

The primary endpoint of this study was the 28-day all-cause mortality rate, and the secondary endpoint was the 360-day all-cause mortality rate.

### 2.4. Statistical analysis

Continuous variables were presented as mean ± standard deviation or median (interquartile range [IQR]) according to data distribution, whereas categorical variables were expressed as proportions. The Kolmogorov–Smirnov test was employed to evaluate the normality of continuous parameters. The analysis of continuous variables was performed using *t* test or ANOVA if they presented a normal distribution and using the Mann–Whitney *U* test or Kruskal–Wallis test if they were non-normal distributions. Kaplan–Meier survival analysis was employed to assess the incidence rate of endpoints among groups based on different levels of the HAR index, and their differences were assessed through log-rank tests.^[18]^ To evaluate influencing factors related to the risk of all-cause death, univariate Cox regression analysis was performed. Cox proportional hazards models were used to calculate the hazard ratio (HR) and 95% confidence interval (CI) between the HAR index and endpoints, and also adjusted for some models (Fig. S1, Supplemental Digital Content, https://links.lww.com/MD/R652).^[19]^ Confounding variables included variables selected based on *P*-value < .05 in univariate analysis. Clinically relevant and prognosis-associated variables were also enrolled in the multivariate model^[20,21]^: model 1: unadjusted; model 2: adjusted for age, gender, race, and weight; and model 3: adjusted for age, gender, race, weight, cardiac valvulopathy, diabetes, hypertension, HF, myocardial infarction, COPD, Hgb, WBC, red blood cell distribution width, potassium, calcium, magnesium, lactate dehydrogenase, glucose, INR, creatinine, Charlson Comorbidity Index, SAPS II, SOFA, HR, RR, temperature, mean noninvasive blood pressure, warfarin, amiodarone, beta-blockers, and NOACs/DOACs. Furthermore, we employed a restricted cubic spline (RCS) regression model with 5 knots to analyze the relationship between baseline HAR index and 28- and 360-day all-cause mortality. The HAR index was entered into the models as continuous variables or ordinal variables (the first quartile of the HAR index was taken as a reference group). The *P* values for trends were calculated using the quartile level. To evaluate the discriminative performance, we calculated the area under the receiver operating characteristic curve of models predicting 28-day mortality based on conventional clinical variables and the SOFA score, with and without including HAR. Further stratified analyses were performed based on gender, age (≤ 60 and >60 years), HF, NOACs/DOACs, warfarin, amiodarone, and beta-blocker, to identify the consistency of the prognostic value of the HAR index for primary outcomes. The interactions between HAR index and variables used for stratification were examined with likelihood ratio tests. A two-sided *P* < .05 was regarded as statistically significant. All statistical analysis was performed by the R software (version 4.0.2; R Foundation for Statistical Computing, Vienna, Austria) and SPSS 22.0 (IBM SPSS Statistics, Armonk). All figures/tables were generated by the authors based on the MIMIC-IV database and have not been published previously.

## 3. Results

### 3.1. Baseline characteristics

In this study, a total of 7801 critically ill patients with AF were enrolled. The median age of the included patients was 75 (IQR: 67–83) years, and 3198 (40.99%) were men. The median HAR index for all included participants was 3.40 (IQR: 2.92–4.00). The 28- and 360-day all-cause mortality rates were 20.11% and 25.87%, respectively (Table 1). Participants were stratified by HAR quartiles – Q1: 2.62 (2.40–2.78), Q2: 3.17 (3.05–3.28), Q3: 3.66 (3.52–3.82), and Q4: 4.55 (4.24–5.06) – yielding 7801 patients (~1950 per group). Baseline characteristics were broadly comparable in age, while the proportion of males declined with higher HAR. Illness severity increased across quartiles (e.g., higher SOFA/SAPS II), accompanied by lower albumin and higher Hgb, and modestly higher heart rate. Anticoagulant/antiarrhythmic use differed by quartile (greater heparin and amiodarone, less warfarin in higher HAR). Short- and longer-term outcomes were graded across HAR: both 28- and 360-day all-cause mortality were highest in Q4. Values are reported as median (IQR) or n (%); group comparisons used appropriate trend tests.

**Table 1:** Characteristics and outcomes of participants categorized by HAR index.*

Categories	Overall	Q1	Q2	Q3	Q4	*P*-value
	N = 7801	N = 1950	N = 1950	N = 1950	N = 1951	
Age (yr)	75.00 (67.00–83.00)	76.00 (67.00–84.00)	76.00 (68.00–83.00)	76.00 (66.00–84.00)	75.00 (66.00–82.00)	**<.001**
Weight (kg)	80.00 (67.05–95.90)	78.50 (66.80–95.00)	80.90 (68.50–95.50)	79.90 (65.60–95.00)	80.60 (67.80–98.00)	**.013**
Sex: male, n (%)	3198.00 (40.99%)	884.00 (45.33%)	788.00 (40.41%)	789.00 (40.46%)	737.00 (37.78%)	**<.001**
Race, n (%)						**<.001**
White	5596.00 (71.73%)	1421.00 (72.87%)	1427.00 (73.18%)	1391.00 (71.33%)	1357.00 (69.55%)	
Black	545.00 (6.99%)	155.00 (7.95%)	141.00 (7.23%)	133.00 (6.82%)	116.00 (5.95%)	
Others	1660.00 (21.28%)	374.00 (19.18%)	382.00 (19.59%)	426.00 (21.85%)	478.00 (24.50%)	
Disease severity score						
SOFA	5.00 (3.00–8.00)	5.00 (3.00–8.00)	5.00 (3.00–7.00)	5.00 (3.00–8.00)	6.00 (4.00–9.00)	**<.001**
SAPS II	41.00 (34.00–51.00)	41.00 (34.00–50.00)	40.00 (32.00–48.00)	40.00 (33.00–50.00)	45.00 (36.00–55.00)	**<.001**
CCI	6.00 (5.00–8.00)	7.00 (5.00–9.00)	6.00 (5.00–8.00)	6.00 (5.00–8.00)	6.00 (4.00–8.00)	**<.001**
Laboratory tests						
Albumin, g/dL	3.00 (2.60–3.40)	3.40 (3.10–3.70)	3.20 (2.80–3.60)	3.00 (2.60–3.30)	2.50 (2.10–2.90)	**<.001**
Hgb, g/dL	10.15 (8.83–11.70)	8.60 (7.80–9.60)	10.00 (8.95–11.20)	10.90 (9.60–12.20)	11.70 (10.20–13.20)	**<.001**
WBC, K/µL	11.30 (8.13–15.70)	9.90 (7.10–14.10)	11.20 (8.30–15.08)	11.60 (8.50–16.10)	12.60 (9.03–17.85)	**<.001**
RDW, vol%	15.10 (13.95–16.90)	15.95 (14.50–18.00)	14.95 (13.83–16.60)	14.70 (13.73–16.40)	14.90 (13.90–16.40)	**<.001**
Sodium, mmol/L	138.00 (135.00–141.00)	138.00 (135.00–141.00)	139.00 (136.00–141.00)	138.00 (135.00–141.00)	138.00 (135.00–141.00)	**.003**
Calcium, mg/dL	8.40 (7.90–8.80)	8.60 (8.10–9.00)	8.40 (8.00–8.90)	8.30 (7.80–8.80)	8.10 (7.50–8.60)	**<.001**
Potassium, mmol/L	4.20 (3.80–4.70)	4.30 (3.90–4.80)	4.20 (3.80–4.60)	4.20 (3.80–4.70)	4.20 (3.70–4.70)	**<.001**
Magnesium, mg/dL	2.00 (1.80–2.30)	2.10 (1.80–2.40)	2.00 (1.80–2.30)	2.00 (1.80–2.20)	2.00 (1.70–2.20)	**<.001**
Lactate dehydrogenase, IU/L	286.00 (215.00–416.00)	282.00 (214.00–403.00)	278.00 (215.00–396.00)	281.00 (210.00–404.00)	305.00 (223.00–479.00)	**<.001**
Glucose, mg/dL	130.00 (106.00–168.00)	129.00 (105.00–166.00)	130.00 (107.00–164.00)	130.00 (106.00–166.00)	133.00 (106.00–175.00)	**.028**
INR	1.40 (1.20–1.80)	1.50 (1.20–2.00)	1.40 (1.20–1.80)	1.40 (1.20–1.70)	1.40 (1.20–1.80)	**<.001**
Creatinine, mg/dL	1.20 (0.80–1.90)	1.30 (0.90–2.30)	1.10 (0.80–1.70)	1.10 (0.80–1.70)	1.20 (0.80–1.90)	**<.001**
HAR index	3.40 (2.92–4.00)	2.62 (2.40–2.78)	3.17 (3.05–3.28)	3.66 (3.52–3.82)	4.55 (4.24–5.06)	**<.001**
Vital signs						
Heart rate, beats/min	88.00 (75.00–105.00)	83.00 (72.00–99.00)	86.00 (74.00–101.00)	89.00 (76.00–105.00)	94.00 (79.00–113.00)	**<.001**
Respiratory rate, times/min	19.00 (16.00–23.00)	19.00 (15.00–23.00)	19.00 (15.00–23.00)	19.00 (16.00–23.00)	20.00 (16.00–24.00)	**<.001**
Saturation of peripheral oxygen, %	97.00 (95.00–100.00)	98.00 (95.00–100.00)	98.00 (95.00–100.00)	97.00 (94.00–100.00)	96.00 (94.00–99.00)	**<.001**
Temperature, °F	98.10 (97.60–98.70)	98.00 (97.60–98.60)	98.10 (97.60–98.70)	98.10 (97.60–98.70)	98.10 (97.60–98.70)	**.010**
NBPm, mm Hg	79.00 (68.00–92.00)	77.00 (67.00–90.00)	79.00 (68.00–92.00)	80.00 (68.00–93.00)	79.00 (68.00–92.00)	**<.001**
Comorbidities						
Valvulopathy, n (%)	1981.00 (25.39%)	651.00 (33.38%)	561.00 (28.77%)	436.00 (22.36%)	333.00 (17.07%)	**<.001**
Diabetes, n (%)	2818.00 (36.12%)	794.00 (40.72%)	718.00 (36.82%)	668.00 (34.26%)	638.00 (32.70%)	**<.001**
Hypertension, n (%)	2892.00 (37.07%)	615.00 (31.54%)	759.00 (38.92%)	780.00 (40.00%)	738.00 (37.83%)	**<.001**
Heart failure, n (%)	4000.00 (51.28%)	1139.00 (58.41%)	1005.00 (51.54%)	960.00 (49.23%)	896.00 (45.93%)	**<.001**
Myocardial infarction, n (%)	983.00 (12.60%)	272.00 (13.95%)	266.00 (13.64%)	228.00 (11.69%)	217.00 (11.12%)	**.015**
COPD, n (%)	1556.00 (19.95%)	419.00 (21.49%)	374.00 (19.18%)	378.00 (19.38%)	385.00 (19.73%)	.255
Medication status						
Insulin, n (%)	4904.00 (62.86%)	1274.00 (65.33%)	1237.00 (63.44%)	1183.00 (60.67%)	1210.00 (62.02%)	**.019**
Heparin, n (%)	7042.00 (90.27%)	1720.00 (88.21%)	1737.00 (89.08%)	1749.00 (89.69%)	1836.00 (94.11%)	**<.001**
Warfarin, n (%)	2507.00 (32.14%)	701.00 (35.95%)	701.00 (35.95%)	615.00 (31.54%)	490.00 (25.12%)	**<.001**
Amiodarone, n (%)	2183.00 (27.98%)	467.00 (23.95%)	537.00 (27.54%)	561.00 (28.77%)	618.00 (31.68%)	**<.001**
Beta-blocker, n (%)	6305.00 (80.82%)	1530.00 (78.46%)	1605.00 (82.31%)	1588.00 (81.44%)	1582.00 (81.09%)	**.016**
NOACs/DOACs, n (%)	1261.00 (16.16%)	292.00 (14.97%)	312.00 (16.00%)	359.00 (18.41%)	298.00 (15.27%)	**.015**
Outcomes						
28-d mortality, n (%)	1569.00 (20.11%)	372.00 (19.08%)	322.00 (16.51%)	366.00 (18.77%)	509.00 (26.09%)	**<.001**
360-d mortality, n (%)	2018.00 (25.87%)	493.00 (25.28%)	433.00 (22.21%)	457.00 (23.44%)	635.00 (32.55%)	**<.001**

CCI = Charlson Comorbidity Index, COPD = chronic obstructive pulmonary disease, HAR = hemoglobin-to-albumin ratio, INR = international normalized ratio, NBPm = mean noninvasive blood pressure, NOACs/DOACs = new oral anticoagulants/direct oral anticoagulants, RDW = red blood cell distribution width, SAPS II = Simplified Acute Physiology Score II, SOFA = Sequential Organ Failure Assessment, WBC = white blood cell count.

* HAR index: Q1: 2.62 (2.40–2.78); Q2: 3.17 (3.05–3.28); Q3: 3.66 (3.52–3.82); Q4: 4.55 (4.24–5.06).

### 3.2. Primary outcomes

**Figure 2.**
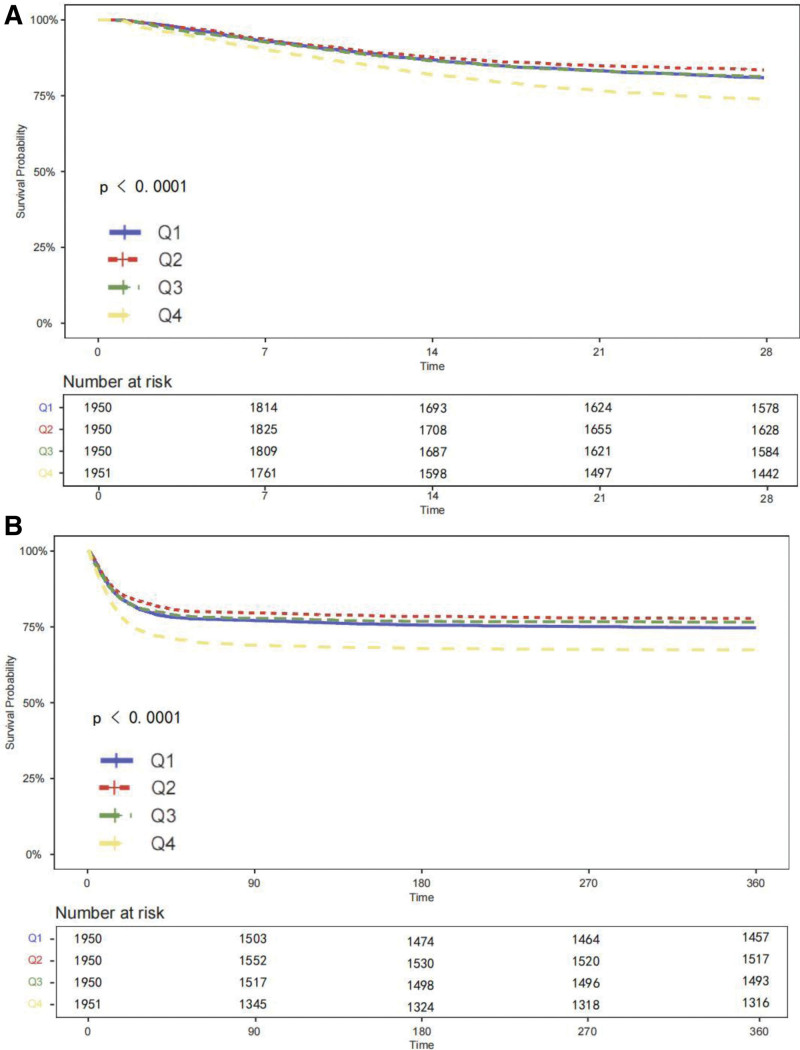
Kaplan–Meier survival curves for all-cause mortality stratified by HAR index quartiles. The curves represent the cumulative probability of all-cause mortality at 28 days (A) and 360 days (B). HAR index quartiles are defined as Q1: 2.62 (2.40–2.78); Q2: 3.17 (3.05–3.28); Q3: 3.66 (3.52–3.82); and Q4: 4.55 (4.24–5.06). HAR = hemoglobin-to-albumin ratio.

Kaplan–Meier curves showed a graded increase in both 28- and 360-day all-cause mortality across HAR quartiles (log-rank *P* < .001 for both; Fig. 2). Table S1, Supplemental Digital Content, https://links.lww.com/MD/R653, presents the results of univariable Cox regression analyses for all-cause mortality in critically ill patients with AF. Variables with *P* < .05 in the univariable analyses, together with clinically relevant factors identified based on clinicians’ judgment and experience, were included as covariates in the multivariable Cox regression models. Results showed that age, weight, WBC, albumin, potassium, magnesium, calcium, creatinine, lactate dehydrogenase, blood glucose, INR, heart rate, RR, body temperature, hypertension, history of COPD, HF, myocardial infarction, valvular heart disease, use of amiodarone, beta-blockers, novel oral anticoagulants, warfarin, and HAR index were significantly associated with all-cause mortality in univariable Cox regression analyses. Cox proportional risk analysis was used to analyze the association between HAR index and 28-day all-cause mortality, and results demonstrated that HAR index was a significant risk factor in the unadjusted model (HR, 1.11 [1.084–1.136] *P* < .001), partly adjusted model (HR, 1.132 [1.102–1.163] *P* < .001) and fully adjusted model (HR, 1.071 [1.034–1.11] *P* < .001) when the HAR index was a continuous variable. When HAR index was a nominal variable, patients in the higher quartile of HAR was significantly associated with higher risk of 28-day all-cause mortality in the 3 established Cox proportional hazards models: unadjusted model (HR, 1.429 [1.25–1.633] *P* < .001), partly adjusted model (HR, 1.444 [1.263–1.651] *P* < .001) and fully adjusted model (HR, 1.307 [1.129–1.491] *P* < .001), compared with subjects in the lowest quartile, and represented a tendency to increase with the HAR index (Table 2).

**Table 2:** Cox proportional hazard ratios (HRs) for 28-day all-cause mortality (models 1–3).

Categories	Model 1			Model 2			Model 3		
	HR (95% CI)	*P*-value	*P* for trend	HR (95% CI)	*P*-value	*P* for trend	HR (95% CI)	*P*-value	*P* for trend
Continuous variable per unit	1.11 (1.084–1.136)	<.001		1.132 (1.102–1.163)	<.001		1.071 (1.034–1.11)	<.001	
Quartile			<.001			<.001			<.001
Q1 (N = 1950) Ref	Ref								
Q2 (N = 1950)	0.858 (0.739–0.996)	.044		0.86 (0.74–0.998)	.047		1.101 (0.944–1.283)	.221	
Q3 (N = 1950)	0.987 (0.854–1.14)	.854		0.988 (0.855–1.142)	.874		1.161 (0.998–1.35)	.053	
Q4 (N = 1951)	1.429 (1.25–1.633)	<.001		1.444 (1.263–1.651)	<.001		1.307 (1.129–1.491)	<.001	

Model 1: unadjusted.

Model 2: adjusted for age, gender, race, and weight.

Model 3: adjusted for age, weight, WBC, albumin, potassium, magnesium, calcium, creatinine, lactate dehydrogenase, blood glucose, INR, heart rate, respiratory rate, body temperature, hypertension, COPD, heart failure, myocardial infarction, valvular heart disease, use of amiodarone, beta-blockers, novel oral anticoagulants, and warfarin.

CI = confidence interval, COPD = chronic obstructive pulmonary disease, INR = INR = international normalized ratio, WBC = white blood cell count.

Similar patterns were observed for 360-day mortality (Table 3). HAR remained an independent predictor of long-term mortality when treated as a continuous variable (fully adjusted HR: 1.07, 95% CI: 1.04–1.11), and patients in the highest HAR quartile had a higher 360-day mortality risk than those in the lowest quartile (fully adjusted HR: 1.32, 95% CI: 1.16–1.50; *P* for trend < .001).

**Table 3:** Cox proportional hazard ratios (HRs) for 360-day all-cause mortality (models 1–3).

Categories	Model 1			Model 2			Model 3		
	HR (95% CI)	*P*-value	*P* for trend	HR (95% CI)	*P*-value	*P* for trend	HR (95% CI)	*P*-value	*P* for trend
Continuous variable per unit	1.01 (1.077–1.127)	<.001		1.12 (1.092–1.148)	<.001		1.073 (1.04–1.107)	<.001	
Quartile			<.001			<.001			<.001
Q1 (N = 1950) Ref	Ref								
Q2 (N = 1950)	0.866 (0.761–0.985)	.028		0.869 (0.764–0.989)	.033		1.126 (0.986–1.286)	.079	
Q3 (N = 1950)	0.927 (0.816–1.052)	.241		0.93 (0.819–1.156)	.263		1.121 (0.982–1.281)	.092	
Q4 (N = 1951)	1.362 (1.21–1.532)	<.001		1.377 (1.224–1.55)	<.001		1.32 (1.161–1.501)	<.001	

Model 1: unadjusted.

Model 2: adjusted for age, gender, race, and weight.

Model 3: adjusted for age, weight, WBC, albumin, potassium, magnesium, calcium, creatinine, lactate dehydrogenase, blood glucose, INR, heart rate, respiratory rate, body temperature, hypertension, COPD, heart failure, myocardial infarction, valvular heart disease, use of amiodarone, beta-blockers, novel oral anticoagulants, and warfarin.

CI = confidence interval, COPD = chronic obstructive pulmonary disease, INR = international normalized ratio, WBC = white blood cell count.

**Figure 3.**
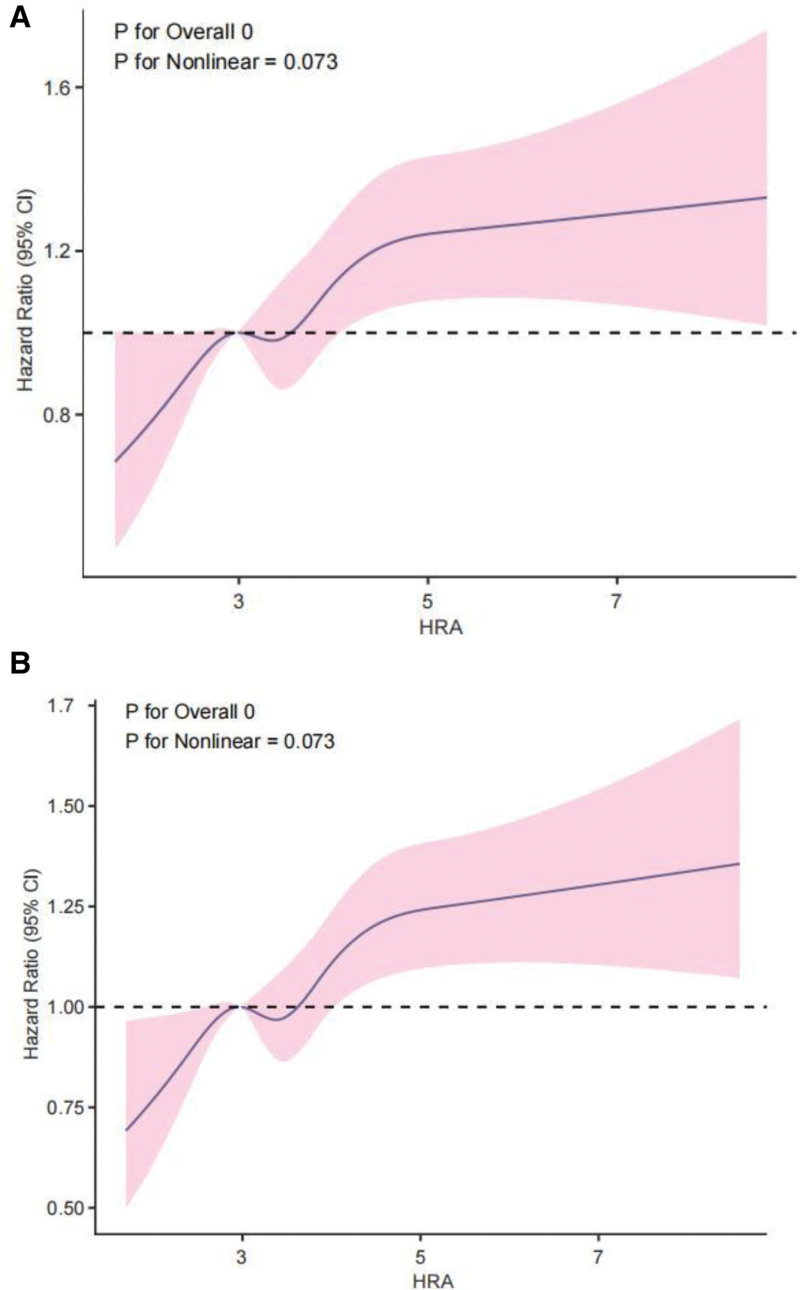
Restricted cubic spline (RCS) analysis of the association between HAR index and mortality. The plots show the approximately linear relationship between the continuous HAR index and the hazard ratio for 28-day (A) and 360-day (B) all-cause mortality. The solid lines represent the hazard ratio estimates, and the shaded areas indicate the 95% confidence intervals. CI = confidence interval, HAR = hemoglobin-to-albumin ratio.

RCS analyses demonstrated an approximately linear relationship between HAR and both 28- and 360-day all-cause mortality, without strong evidence of nonlinearity (Fig. 3).

In evaluating the validation of HAR effect, we added HAR to the SOFA score and compared the SOFA and SOFA + HAR models. For the 28-day outcome, the addition of HAR showed a modest improvement in the SOFA score, with statistically significant results. The area under the receiver operating characteristic curve increased from 0.686 to 0.699 (DeLong test, *P* < .0001, Fig. S2, Supplemental Digital Content, https://links.lww.com/MD/R652).

### 3.3. Subgroup analysis

**Figure 4.**
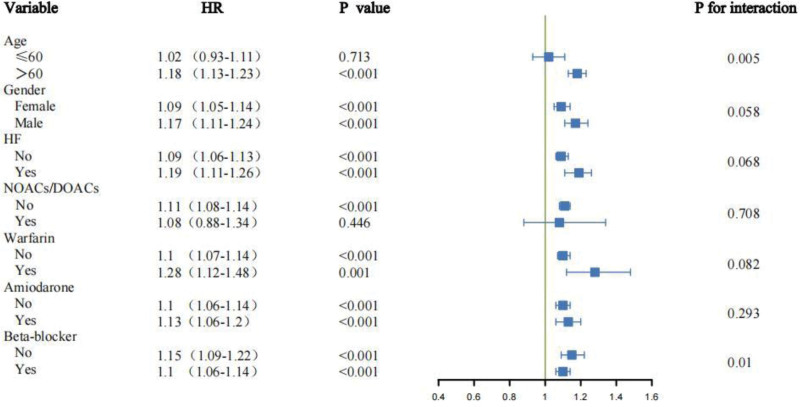
Forest plot of subgroup analyses for 28-day all-cause mortality. Hazard ratios and 95% confidence intervals for the association between HAR and short-term mortality are shown across clinical strata, including age, gender, heart failure history, and medication use. Significant interactions were observed for age and beta-blocker use. HAR = hemoglobin-to-albumin ratio, HF = heart failure, HR = hazard ratio, NOACs/DOACs = new oral anticoagulants/direct oral anticoagulants.

Across most predefined subgroups, higher HAR was consistently associated with increased 28-day mortality (HRs > 1, *P* < .001). Significant interactions were observed for age (*P*_interaction = .005) and beta-blocker use (*P*_interaction = .01): the effect was stronger in patients >60 years (HR: 1.18, 95% CI: 1.13–1.23) versus ≤60 years (HR: 1.02, 95% CI: 0.93–1.11), and in those not using beta-blockers (HR: 1.15, 95% CI: 1.09–1.22) versus users (HR: 1.10, 95% CI: 1.06–1.14). No significant interactions were detected for gender, HF, warfarin, amiodarone, or NOACs/DOACs (all *P* > .05; Fig. 4).

**Figure 5.**
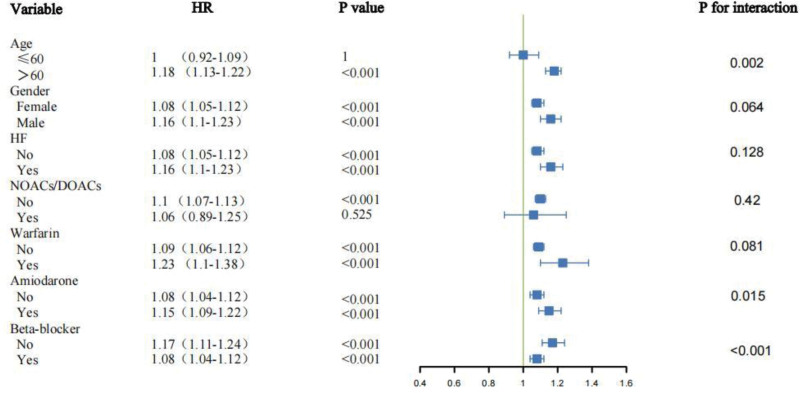
Forest plot of subgroup analyses for 360-day all-cause mortality. This plot evaluates the consistency of the prognostic value of HAR for long-term mortality across subgroups. Significant interactions emerged for age, beta-blocker use, and amiodarone therapy. HAR = hemoglobin-to-albumin ratio, HF = heart failure, HR = hazard ratio, NOACs/DOACs = new oral anticoagulants/direct oral anticoagulants.

At 360 days, HAR remained predictive of mortality with similar effect modification by age (*P*_interaction = .002) and beta-blocker use (*P*_interaction < .001). Additionally, amiodarone therapy emerged as a new modifier (*P*_interaction = .015), with stronger risk in users (HR: 1.15, 95% CI: 1.09–1.22) compared with nonusers (HR: 1.08, 95% CI: 1.04–1.12). Other subgroup factors showed no significant interaction (Fig. 5).

## 4. Discussion

In this large-scale retrospective cohort of 7801 critically ill patients with AF from the MIMIC-IV database, we found that a higher HAR was independently associated with an increased risk of both short-term (28-day) and long-term (360-day) all-cause mortality. The relationship was consistent across multiple models after adjusting for demographics, comorbidities, laboratory parameters, vital signs, disease severity scores, and medications. RCS analyses demonstrated an approximately linear association without evidence of nonlinearity, and Kaplan–Meier curves confirmed a graded increase in mortality with rising HAR levels. In exploratory analysis, HAR modestly improved the model discrimination for short-term mortality based on SOFA scores.

Subgroup analyses further revealed that the prognostic effect of HAR was more pronounced in older patients and those not receiving beta-blockers, and a significant interaction emerged with amiodarone use at the 360-day follow-up. This outcome reflects medium-to-long-term risks. Amiodarone is known for its various long-term side effects, which may synergize with the pathophysiological conditions (malnutrition, inflammation) represented by HAR, exacerbating long-term mortality risk. This results in significantly higher long-term mortality risk for patients taking amiodarone compared with nonusers when HAR is high. It is worth considering that HAR cannot only predict short-term outcomes but may also identify patient groups at higher risk due to the side effects of long-term medication (such as amiodarone). These findings highlight HAR as a robust and easily obtainable biomarker for risk stratification in AF patients admitted to intensive care.

Previous studies have primarily examined HAR in oncological and surgical populations.^[22]^ For example, HAR has been reported as an independent prognostic factor in colorectal cancer, gastric cancer, and nasopharyngeal carcinoma, reflecting the combined impact of anemia and malnutrition on survival outcomes.^[13,14]^ More recently, evidence has begun to emerge on the role of nutrition- and inflammation-based indices in cardiovascular disease prognosis, such as the modified Glasgow Prognostic Score.^[23,24]^ However, to our knowledge, this is the first study systematically investigating HAR in a large AF cohort within the critical care setting. Our results extend the scope of HAR beyond oncological and surgical conditions, linking it with arrhythmia-related outcomes and reinforcing the importance of systemic metabolic and inflammatory status in cardiovascular prognosis.

Theoretically, HAR integrates 2 clinically important dimensions: Hgb levels reflect systemic oxygen-carrying capacity, and anemia is strongly associated with myocardial hypoxia, activation of the sympathetic nervous system, and renin-angiotensin-aldosterone system – all of which contribute to atrial structural and electrophysiological remodeling and perpetuation of AF.^[25,26]^ Conversely, elevated Hgb may increase blood viscosity and thrombotic risk, thereby enhancing atrial stasis and systemic embolic events.^[27]^ Albumin, on the other hand, is not only a marker of nutritional reserve but also exerts anti-inflammatory, antioxidant, and anticoagulant effects.^[28]^ Hypoalbuminemia reflects a state of systemic inflammation, increased vascular permeability, and protein catabolism, which are frequently observed in critical illness.^[29]^ Reduced albumin levels may impair endothelial function, amplify oxidative stress, and promote platelet aggregation, thereby aggravating both thrombotic complications and mortality in AF.^[30]^ Accordingly, a high HAR may represent a deleterious phenotype characterized by the co-existence of elevated Hgb and reduced albumin.^[18,19,31]^ Hypoalbuminemia has been associated with gut microbiota dysbiosis and immune-metabolic alterations, which may drive atrial electrical remodeling and fibrosis through systemic inflammatory mediators.^[32]^ Similarly, anemia and disordered iron metabolism may exacerbate mitochondrial dysfunction and myocardial energy derangements, thereby accelerating atrial remodeling and increasing vulnerability to arrhythmic and ischemic events.^[33,34]^

Our findings should also be interpreted in the context of existing risk stratification tools. Traditional scores such as CHA_2_DS_2_-VASc and HAS-BLED have been widely applied to predict thromboembolic or bleeding risk in patients with AF, whereas severity indices such as SOFA or SAPS II are routinely used in the ICU setting. However, these models were not designed to capture the prognostic impact of nutritional and inflammatory status, and their predictive ability for all-cause mortality remains modest. Recent studies have demonstrated that composite indices derived from routine blood counts – such as the neutrophil-to-lymphocyte ratio, systemic immune-inflammation index, and neutrophil percentage-to-albumin ratio – also carry significant prognostic information in AF and HF populations.^[35–37]^ Within this framework, HAR provides a simple but biologically plausible metric that integrates hematologic and nutritional-inflammatory pathways. The potential incremental value of HAR in combination with established scores warrants future evaluation.

Several limitations should be acknowledged. First, the retrospective nature of our study and reliance on a single-center database (MIMIC-IV) limit causal inference and generalizability. Second, although we adjusted for a wide range of demographic, clinical, and severity variables, residual confounding from unmeasured factors (e.g., iron status, C-reactive protein, or other inflammatory biomarkers) cannot be excluded.^[38]^ Our reporting follows established guidelines for observational studies to mitigate some of these concerns.^[39]^ Third, HAR was calculated only at baseline, and dynamic changes during hospitalization were not captured. Fourth, the biological mechanisms linking HAR with AF-related mortality remain speculative, requiring mechanistic and translational research. In addition, “critically ill” status in this study was pragmatically defined by ICU admission rather than by a specific SOFA cutoff. While this approach reflects real-world clinical triage and is consistent with prior MIMIC-IV analyses, it may introduce some heterogeneity in illness severity and should be considered when interpreting our findings. Looking forward, prospective validation in multicenter cohorts will be essential to confirm the robustness and generalizability of our findings. Beyond static measurements at admission, longitudinal assessment of HAR trajectories may capture dynamic shifts in inflammatory and nutritional status and improve risk prediction. Moreover, whether therapeutic strategies – such as tailored nutritional support, anti-inflammatory interventions, or optimization of medication regimens – can mitigate the adverse prognostic impact of elevated HAR represents an important clinical question. Ultimately, integration of HAR into multimodal prognostic models, alongside conventional clinical variables and emerging omics-based biomarkers, could refine individualized risk stratification and inform precision management of patients with AF in critical care.

## 5. Conclusion

In conclusion, this study demonstrated that an elevated HAR was independently associated with increased short- and long-term mortality in critically ill patients with AF. As a simple and routinely available biomarker, HAR may provide incremental value for risk stratification beyond conventional clinical parameters. Further prospective, multicenter, and mechanistic studies are needed to validate these findings and to clarify whether interventions targeting nutritional and inflammatory status could improve outcomes in this high-risk population.

## Author contributions

**Conceptualization:** Yinghan Sun.

**Data curation:** Yinghan Sun.

**Formal analysis:** Yinghan Sun.

**Methodology:** Yuxiao Zhai.

**Visualization:** Yuxiao Zhai.

**Writing – original draft:** Chunyang Lv.

**Writing – review & editing:** Hongyi Cai.

## Supplementary Material




