# Supplementary material for: The prognostic value of hemoglobin-to-albumin ratio in critically ill patients with atrial fibrillation: A retrospective cohort study
Source: Medicine (Baltimore). 2026 Apr 24;105(17):e48211. doi: 10.1097/MD.0000000000048211 (PMC13124338; doi:10.1097/MD.0000000000048211)
Supplement: Supplementary file 2 [file medi-105-e48211-s002.pdf]

COX Regression HR Forest Plot

Characteristics	Number(%)	HazardRatio(HR)	Pvalue
age	74.27 (11.90)	1.012 (1.008,1.016)	<0.001
albumin	3.03 (0.61)	0.594 (0.547,0.645)	<0.001
amiodarone			
0	5618 (72.0)		
1	2183 (28.0)	1.418 (1.278,1.573)	<0.001
bblocker			
0	1496 (19.2)		
1	6305 (80.8)	0.428 (0.385,0.476)	<0.001
Ca	8.35 (0.86)	0.911 (0.859,0.965)	0.002
charlson	6.61 (2.71)	1.113 (1.093,1.132)	<0.001
copd			
0	6245 (80.1)		
1	1556 (19.9)	1.179 (1.047,1.327)	0.007
Cr	1.71 (1.55)	1.117 (1.09,1.144)	<0.001
diabetes			
0	4983 (63.9)		
1	2818 (36.1)	0.98 (0.884,1.087)	0.7
gender			
F	3198 (41.0)		
M	4603 (59.0)	0.917 (0.83,1.014)	0.091
GLu	149.27 (77.87)	1.001 (1.001,1.002)	<0.001
heparin			
0	759 (9.7)		
1	7042 (90.3)	1.011 (0.852,1.199)	0.902
hf			
0	3801 (48.7)		
1	4000 (51.3)	1.117 (1.011,1.233)	0.029
HGB	10.39 (2.04)	0.94 (0.917,0.964)	<0.001
HR	91.22 (23.02)	1.007 (1.004,1.009)	<0.001
HRA	3.55 (1.01)	1.11 (1.084,1.136)	<0.001
HRA_EqualSizeBin			
1	1950 (25.0)		
2	1950 (25.0)	0.858 (0.739,0.996)	0.044
3	1950 (25.0)	0.987 (0.854,1.14)	0.854
4	1951 (25.0)	1.429 (1.25,1.633)	<0.001
htn			
0	4909 (62.9)		
1	2892 (37.1)	0.811 (0.73,0.901)	<0.001
INR	1.76 (1.18)	1.117 (1.087,1.148)	<0.001
insulin			
0	2897 (37.1)		
1	4904 (62.9)	0.998 (0.901,1.106)	0.968
K	4.29 (0.79)	1.154 (1.088,1.224)	<0.001
id	482.08 (953.35)	1 (1,1)	<0.001
Mg	2.07 (0.61)	1.07 (1.015,1.128)	0.012
mi			
0	6818 (87.4)		
1	983 (12.6)	1.274 (1.11,1.463)	0.001
Na	138.02 (5.68)	0.995 (0.987,1.004)	0.3
nbpm	80.97 (19.00)	0.995 (0.993,0.998)	0.001
noacsdoacs			
0	6540 (83.8)		
1	1261 (16.2)	0.349 (0.287,0.425)	<0.001
race			
1	5596 (71.7)		
2	545 (7.0)	1.113 (0.916,1.351)	0.28
3	1660 (21.3)	1.369 (1.221,1.534)	<0.001
RDW	15.69 (2.47)	1.125 (1.107,1.144)	<0.001
RR	19.84 (6.47)	1.03 (1.023,1.036)	<0.001
sapsii	43.31 (13.63)	1.043 (1.04,1.047)	<0.001
sofa	5.89 (3.67)	1.17 (1.156,1.183)	<0.001
SpO2	97.76 (110.60)	1 (1,1)	0.18
temperaturef	97.99 (3.43)	0.987 (0.977,0.997)	0.009
valve			
0	5820 (74.6)		
1	1981 (25.4)	0.839 (0.745,0.944)	0.004
VKA			
0	5294 (67.9)		
1	2507 (32.1)	0.262 (0.226,0.305)	<0.001
WBC	13.06 (9.82)	1.01 (1.007,1.012)	<0.001
weight	83.63 (24.97)	0.998 (0.996,1)	0.024

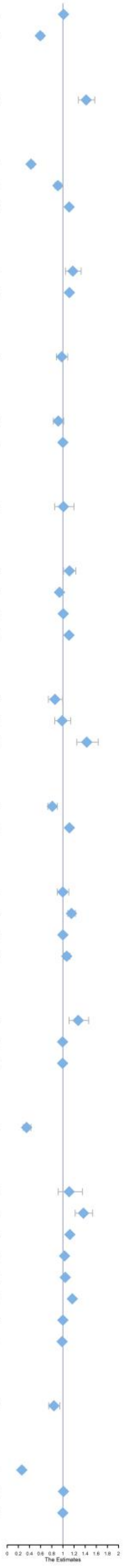

Supplementary Figure S1. Univariable Cox regression forest plot.

Forest plot of hazard ratios (HRs) with 95% confidence intervals (CIs) from univariable Cox proportional hazards models for all-cause mortality in critically ill patients with atrial fibrillation. Reference categories are indicated where applicable; points represent HR estimates and horizontal lines represent 95% CIs.

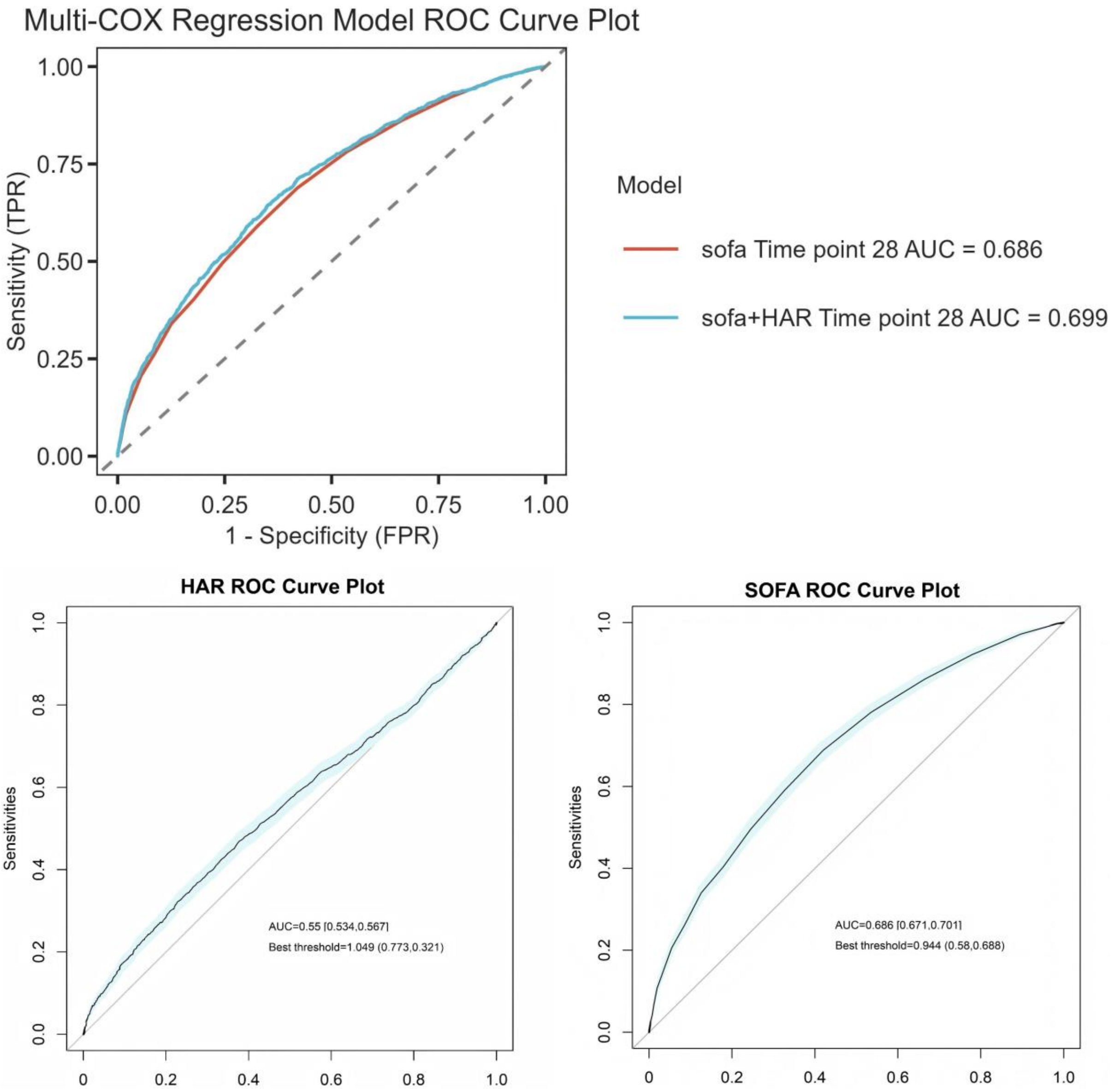

Supplementary Figure S2. ROC curves for prediction of 28-day mortality (SOFA vs. SOFA+HAR).

Receiver operating characteristic (ROC) curves comparing the discriminative performance of the SOFA-only model and the SOFA+HAR model for 28-day all-cause mortality. Adding HAR to SOFA yielded a modest but statistically significant improvement in discrimination (AUC 0.686 vs. 0.699; DeLong test $p < 0.0001$). Additional panels show the ROC curves of HAR alone (AUC = 0.55, 95% CI 0.534–0.567) and SOFA alone (AUC = 0.686, 95% CI

0.671–0.701), with the best thresholds and corresponding sensitivity/specificity as displayed in the figure.
